# GSK3**β** guides chromosomal repair pathway selection to support BRCA1-independent PARP inhibitor sensitivity

**DOI:** 10.1172/JCI197910

**Published:** 2025-11-17

**Authors:** Justin W. Leung, David Gius

**Affiliations:** 1 Department of Radiation Oncology, Mays Cancer Center at University of Texas Health San Antonio MD Anderson, Joe R. and Teresa Lozano Long School of Medicine, San Antonio, Texas, USA.; 2 Barshop Institute for Longevity and Aging Studies, University of Texas Health San Antonio, San Antonio, Texas, USA.

## Abstract

Glycogen synthase kinase-3β (GSK3β) is an established regulator in the DNA double-strand break (DSB) repair pathway. Recent work by Allam et al. revealed a mechanism of DSB repair pathway choice through GSK3β-mediated, site-specific phosphorylation of the tumor suppressor p53 binding protein 1 (53BP1) at threonine 334 (T334). 53BP1 T334 phosphorylation prevented interaction between 53BP1 and its downstream functional partners, PTIP and RIF1, thereby inhibiting 53BP1-directed nonhomologous end joining (NHEJ). Additionally, 53BP1 T334 phosphorylation promoted recruitment of CtIP and RPA32 to DNA damage sites to facilitate homologous recombination (HR). In contrast with loss of 53BP1 function, a 53BP1 T334A phospho-deficient mutant accumulated aberrantly at DSBs, where it impaired end resection and suppressed HR activity. These surprising results suggest that GSK3β may select between NHEJ and HR DNA repair pathways. Additionally, these data support targeting the GSK3β/53BP1 axis to enhance PARP inhibitor efficacy in solid tumors, regardless of BRCA1 status.

BRCA1/2 mutations confer a large increase in risk of developing breast and ovarian cancer, as well as increase the risk of pancreatic, prostate, and other cancers. Tumors harboring loss-of-function mutations in BRCA1 or BRCA2 are deficient in homologous repair (HR), one of two main pathways to repair DNA double-strand breaks (DSBs), making these tumors dependent on PARP1-mediated repair of single-strand breaks and stalled replication forks. Inhibition of PARP1 in these cells leads to unrepaired DNA lesions, replication fork collapse, and the accumulation of lethal DSBs. This mechanistic insight has been successfully translated into targeted therapy with PARP inhibitors (PARPi), which are now approved for BRCA1/2-mutant breast, ovarian, pancreatic, and prostate cancers (1, 2).

However, the binary classification of tumors as “BRCA-deficient” or “BRCA-proficient” is increasingly recognized as an oversimplification. A subset of BRCA-proficient tumors responds to PARPi, whereas some BRCA-deficient tumors display intrinsic or acquired resistance. This discrepancy points to a more complex landscape of HR regulation, in which additional factors can modulate pathway choice and PARPi sensitivity independently of BRCA1/2 status.

## DNA DSB repair pathway choice

DNA DSBs are among the most cytotoxic forms of DNA damage, and their accurate repair is essential for preserving genomic stability and preventing tumorigenesis. Cells have evolved two main pathways to repair DSBs: nonhomologous end joining (NHEJ), which ligates DNA ends with minimal processing, and HR, which uses a homologous DNA template to restore the original sequence. The decision between these pathways is a central point of regulation in the DNA damage response (DDR), influencing not only cell fate but also therapeutic sensitivity in cancer (3).

The commitment to HR is initiated by 5′-to-3′ end resection, generating 3′ single-stranded DNA overhangs. This step is tightly regulated by the MRN complex (MRE11-RAD50-NBS1), CtIP, and accessory nucleases such as EXO1 and DNA2, often under the control of checkpoint kinases ATM and ATR. Once resection has occurred, the single-stranded DNA is rapidly coated by replication protein A (RPA), which is subsequently displaced by RAD51 recombinase through the action of the BRCA1-PALB2-BRCA2 complex. RAD51 filament assembly is the defining step of HR, enabling the search for homology and strand invasion. In the absence of efficient resection or RAD51 loading, DSB repair is diverted toward NHEJ or alternative end joining, both of which are inherently more error prone (4, 5).

A critical regulator of DSB repair pathway choice is the DDR mediator 53BP1 (p53 binding protein 1), which functions as a barrier to end resection, thereby favoring repair by NHEJ over HR, particularly in G1 phase when a sister chromatid is absent. 53BP1 achieves NHEJ choice through recruitment of downstream effectors such as RIF1 and the shieldin complex, which protect DNA ends from nucleolytic degradation. In S/G2 phase, BRCA1 antagonizes 53BP1 to allow resection and commit the break to HR (6–8). Loss of 53BP1 can partially restore HR in BRCA1-deficient cells, a phenomenon with direct clinical implications for PARPi resistance. Thus, the dynamic interplay between BRCA1 and 53BP1 is a major determinant of DSB repair outcome and therapeutic response.

## Kinases as regulators of DSB repair pathway choice

**Figure 1:**
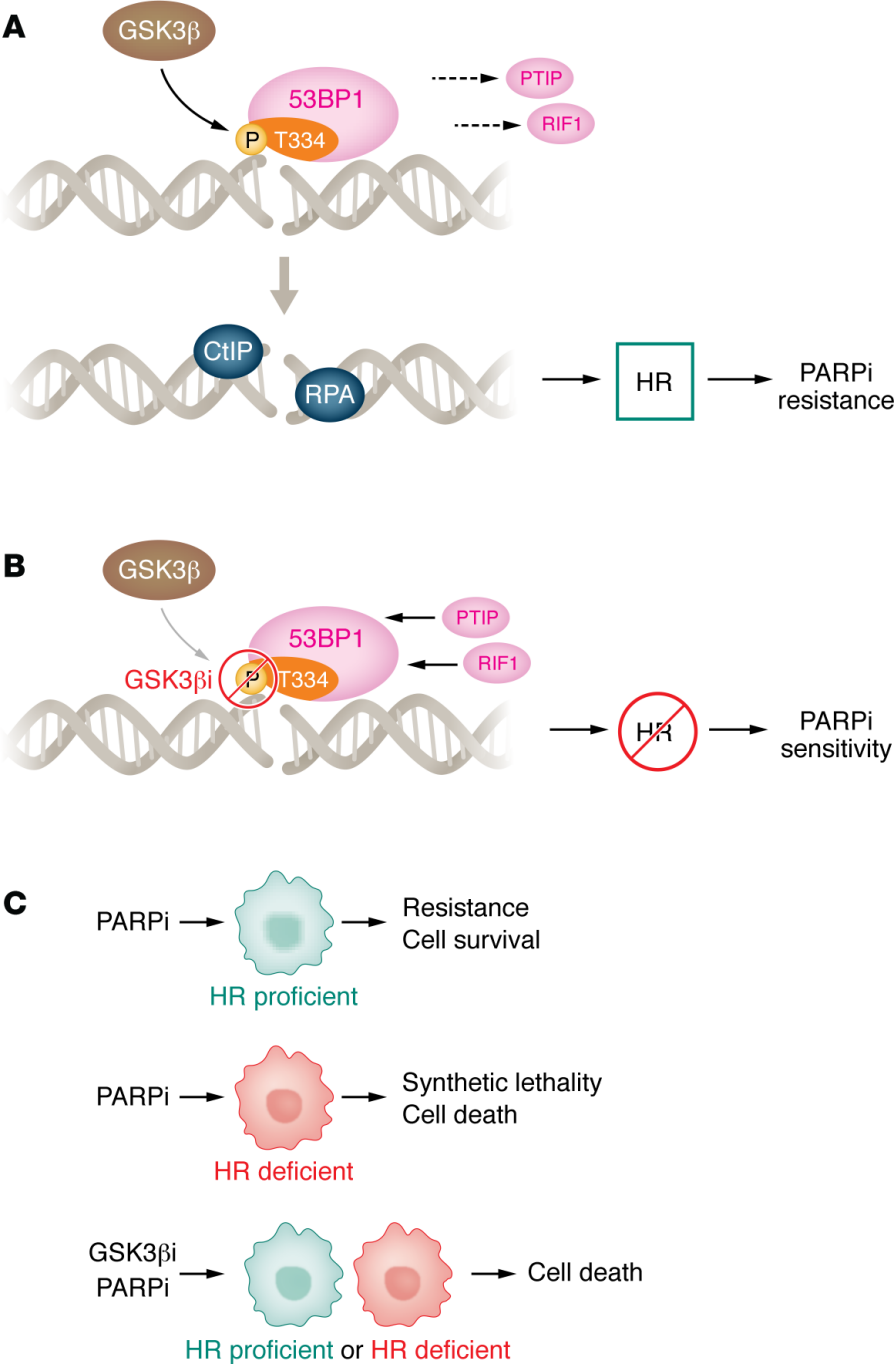
GSK3β-guided selection of DNA double stranded break pathways affects PARP inhibitor sensitivity. (A) Allam et al.’s study described the noncanonical mechanistic regulation of GSK3β-mediated 53BP1 phosphorylation at DSBs. 53BP1 phosphorylation prevented its interaction with PTIP and RIF1 and recruited CtIP and RPA to the DSB, promoting homologous recombination (HR) and therefore resistance to PARP inhibition (PARPi). (**B**) A GSK3β inhibitor (GSK3βi) sensitized HR-proficient cells to PARPi by blocking the noncanonical regulation shown in panel A. (**C**) Summary of therapeutic outcomes in HR-proficient and -deficient cells in response to PARPi or PARPi/GSK3βi.

While DDR signaling is widely recognized to depend on PI3K-like kinases (ATM, ATR, and DNA-PKcs) and checkpoint kinases (CHK1 and CHK2), there is a growing appreciation that other cellular kinases influence repair pathway usage (9). In the current issue of JCI, Allam and colleagues (10) presented compelling evidence that glycogen synthase kinase-3β (GSK3β) acts as a key determinant of DNA DSB repair pathway choice via phosphorylating 53BP1 at threonine 334 (T334) following DNA damage (Figure 1A). Interestingly, unlike the canonical ATM-mediated phosphorylation, which enhances 53BP1’s ability to protect DNA ends and drives NHEJ, phosphorylation at T334 attenuated this function. The authors demonstrated that GSK3β-mediated 53BP1 T334 phosphorylation weakened the association between 53BP1 and its NHEJ cofactors RIF1 and PTIP. This disruption allowed resection machinery to engage the DNA ends, facilitating the recruitment of HR proteins such as CtIP, RPA32, BRCA1, and RAD51 (10).

Allam et al. showed that the kinetics of this phosphorylation were consistent with HR engagement: phospho-T334 foci accumulated later after DNA damage, in line with the slower timing of HR repair rather than the rapid response typical of NHEJ. Loss of T334 phosphorylation, either through a T334A substitution or pharmacologic inhibition of GSK3β, resulted in prolonged retention of 53BP1 at DSB sites, an increase in NHEJ activity, and a reduction in HR efficiency, even in cells with functional BRCA1 (Figure 1, A and B). This work therefore defines T334 phosphorylation as a negative regulatory event for 53BP1’s end-protection role, functioning as a molecular switch to tip the balance toward HR.

## Therapeutic implications: expanding the reach of PARP inhibitors

The GSK3β-mediated mechanism of DSB repair pathway choice has considerable translational relevance. In multiple cell line models, loss of 53BP1 T334 phosphorylation produced an homologous recombination deficiency-like state that rendered tumors hypersensitive to PARPi, regardless of BRCA1 status. Both the T334A mutation and pharmacologic inhibition of GSK3β enhanced the cytotoxic effects of the PARPi Olaparib in vitro. In orthotopic and subcutaneous mouse models, GSK3β inhibitors in combination with Olaparib substantially reduced tumor growth in both BRCA1-proficient and -deficient settings. Notably, this synergy required functional 53BP1, underscoring the specificity of the pathway.

By demonstrating that modulation of 53BP1 phosphorylation can convert HR-proficient tumors into an HR-deficient state (Figure 1B), this work suggests a strategy to deliberately broaden the population of patients who might benefit from PARPi therapy. The approach selectively reprograms DNA repair pathway choice without abolishing NHEJ entirely, potentially mitigating the systemic toxicity that can accompany more global suppression of DNA repair.

Importantly, GSK3β is a well-studied, druggable kinase, and inhibitors have already been tested in the clinic for conditions ranging from Alzheimer’s disease to cancer (11–13). This positions GSK3β inhibition as an attractive and potentially rapid route to translation, with the possibility of repurposing existing agents as PARPi sensitizers. Combining GSK3β inhibitors with PARPi could expand indications to HR-proficient tumors, overcome resistance driven by HR restoration, and fine-tune DNA damage responses in a way that maximizes therapeutic efficacy while avoiding broad suppression of repair capacity.

## Future directions

While the data presented in this manuscript is of mechanistic importance, several important questions remain. The upstream signals that direct GSK3β to phosphorylate 53BP1 after DNA damage are unknown, as are the cell cycle or chromatin contexts in which this modification is most active. It is also unclear whether T334 phosphorylation influences other aspects of 53BP1 function, such as replication fork stability, alternative end joining, or checkpoint signaling. From a precision oncology standpoint, identifying tumor genotypes that predict responsiveness to GSK3β-PARPi combinations will address an important unmet clinical need (Figure 1C). Additionally, the potential for synergy with other DNA damaging agents, such as radiation, topoisomerase inhibitors, or ATR/CHK1 inhibitors, warrants investigation.

## Conclusion

The identification of GSK3β-mediated phosphorylation of 53BP1 at T334 as a determinant of DSB repair pathway choice, reframes our understanding of 53BP1 regulation (Figure 1, A and B). By fine tuning 53BP1’s activity, GSK3β shifts the balance between NHEJ and HR, creating a therapeutically exploitable vulnerability (Figure 1C). With GSK3β inhibitors already in the clinical pipeline, the translation of these findings into combinatorial trials with PARPi could be fast tracked. If successful, such strategies could not only extend the reach of PARPi therapy but also overcome resistance in HR-proficient tumors, representing a formidable advance in the precision targeting of DNA repair in cancer (Figure 1C).

## Funding support

NCI grants R01CA257148, R01CA214025 (DG).
The Cancer Prevention and Research Institute of Texas (CPRIT) grant, RR20012 (DG).
NIGMS R35GM137798-01 (JL).
NCI R01CA244261-01A1 (JL).
American Cancer Society RSG-20-131-01-DMC (JL).
University of Texas STARs award (JL).
NCI Cancer Center Support Grant P30CA054174 (The Mays Cancer Center).
The Sam and Ann Barshop Institute of Longevity.
Aging Studies and the San Antonio Nathan Shock Center.

## Version 1 11/17/2025

Electronic publication
